# Supplementary material for: Spontaneous formation of structurally diverse membrane channel architectures from a single antimicrobial peptide
Source: Nat Commun. 2016 Nov 22;7:13535. doi: 10.1038/ncomms13535 (PMC5121426; doi:10.1038/ncomms13535)
Supplement: Supplementary Information — Supplementary Figures 1-2, Supplementary Tables 1-3 [file ncomms13535-s1.pdf]

Supplementary Data

Supplementary Figure 1 – Structural assembly motifs and hydrogen bond formation network

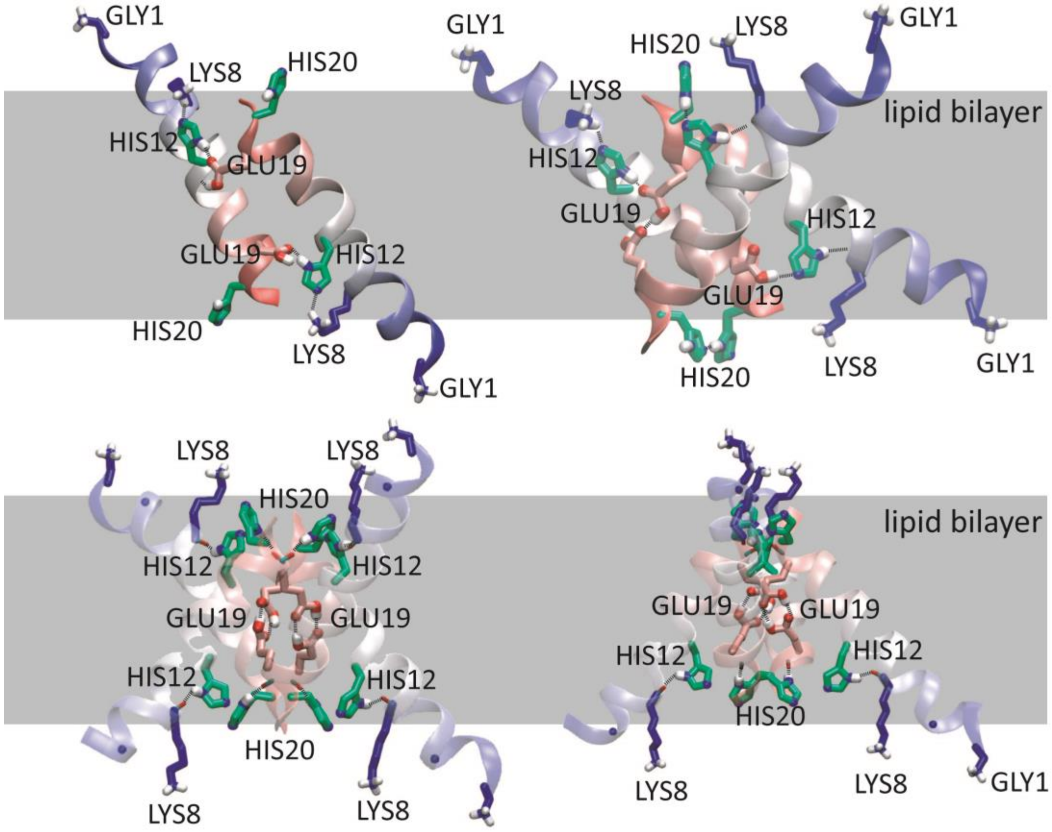

Supplementary Figure 1 | Structural assembly motifs and hydrogen bond network. Hydrogen bond network in the maculatin oligomers (P15A mutant), shown here for some low order oligomers, involve a number of charged, polar, and aromatic residues. The recurring structural motif of the most populated clusters in all simulations is an anti-parallel dimer offset by 8-10 residues. This enables a tightly woven, heterogeneous hydrogen bonding network between the pore lining His12, Gln19, and His20 residues, while the exposed N-terminal parts of the peptides, which include the two cationic groups (Gly1, Lys8) point high into the polar and solvated bilayer interface. Larger oligomers are symmetric combinations of this basic dimer motif. Low level oligomers (dimer, trimer, tetramer, and pentamer) do not form a central water-filled pore. Water molecules rarely penetrate these assemblies as all polar sidechains have saturated their hydrogen bonding capacity by bonding with neighbouring or opposing helices. Water pores form only in higher order oligomers (hexamer, heptamer, and octamer), where inter-helical hydrogen bonds of side chains are reduced in favour of bonds with a central water-filled pore. Interhelical hydrophobic contacts do not play a major role in pore stability, contrary to typical integral membrane protein channels that are dominated by interhelical hydrophobic contacts. Many other possible arrangements, including parallel assemblies exist, but none are as populated as the anti-parallel ones shown here.

Supplementary Figure 2 – Mechanisms of ion conduction for low order oligomers

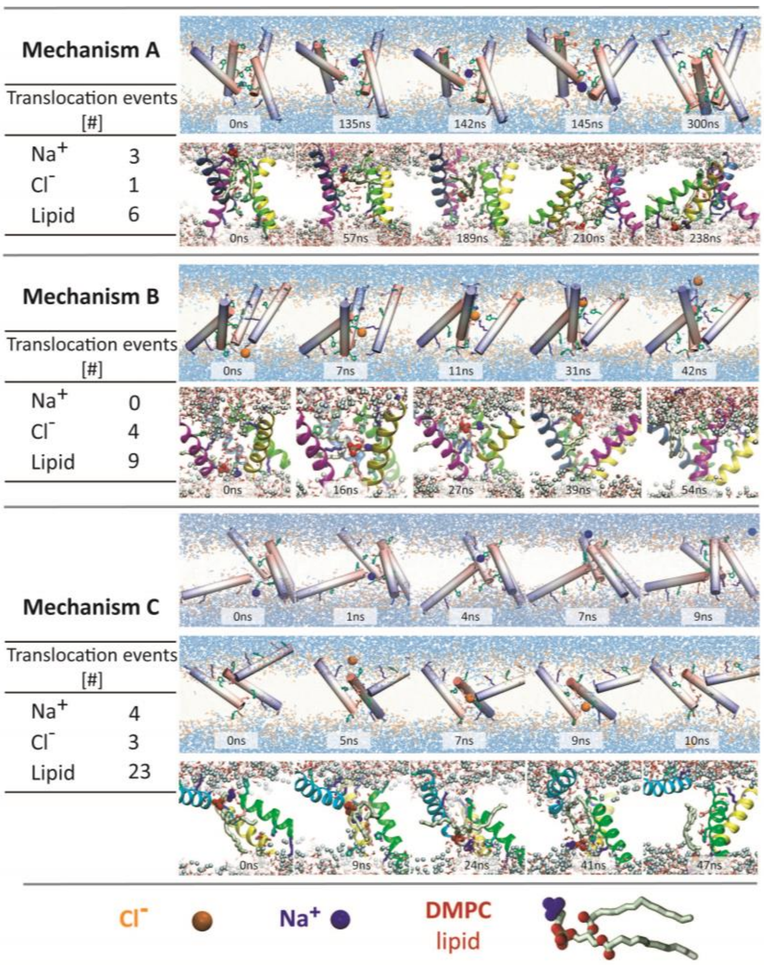

Supplementary Figure 2 | Mechanisms of ion conduction for low order oligomers. Maculatin peptides can directly assist ions or lipids transmission through the membrane or form low level transmembrane oligomers in which ions can translocate using channel-like mechanisms. There is a clear distinction between cation (Na^+) and anion (Cl^-) translocation. **a**, Na^+ conductance occurs via an opening and closing mechanism. The dry, antiparallel tetramer pore opens to form two dimers, creating a large water-filled pore lined with polar sidechains, through which the Na^+ and DMPC were found to translocate. After conduction the pore closes and becomes dry again. **b,** Cl^- conductance is fundamentally different, in that a large water filled pore is not required. The Lys8 sidechain plays a crucial role in pulling and carrying the anion from the lipid headgroup region into the inner, almost dry pore. The symmetric arrangement of Lys sidechains from different peptides inside the pore acts as a ladder for the anions and helps in translocating these across the membrane. **c,** Both cations and anions are able to translocate the membrane using a 'terminal brush' mechanism, that requires no ordered pore, and involves very few water molecules. The ion is caught by the polar terminus of a surface bound peptide (N-terminus for Cl^- and phosphate group of lipid, C-terminus for Na^+ and choline group of lipid), and cornered against the background surface of other TM-inserted peptides. The surface bound peptide then completes a brush-like tilting motion, sweeping the ion across to the other side, and returning to its original surface bound state after completing its motion. The whole process is very fast (10 ns), can occur for Na^+, Cl^-, and lipids and requires no TM voltage. Because large conformational changes of oligomers are involved in all mechanisms, which always entail crossing a considerable free energy barrier, the rate of ion permeation events via these mechanisms is very low (~10^5 s^{-1}). The flux data in the left side of the figure are calculated from simulations DM1 and DM2.

Supplementary Table 1 – List of molecular dynamics simulations performed

**Simulation**	**T** [°C]	**Length** [µs]	**Peptides / Lipid**	**Lipid**	**Mutations**	**Na/Cl** [mM]
DM1	150	50	1 / 30	DMPC	P15A	165
DM2	150	10	1 / 12.5	DMPC	P15A,E19Q	165
DM3	150	10	1 / 12.5	DMPC	E19Q	165
DP1†	150	8	1 / 12.5	DPPC	WT	165
DP2	150	6	1 / 12.5	DPPC	WT	165
DP3	150	22	1 / 12.5	DPPC	P15A,E19Q	165
DP4	150	21	1 / 12.5	DPPC	E19Q	165

Supplementary Table 1 | List of molecular dynamics simulations performed. All systems have neutralized glutamic acid, except DP1†, which has a charged Glu. The total simulation time was 127 µs.

Supplementary Table 2 – Conduction properties of spontaneously formed maculatin channel-like pores

Channel-like Pore*	System Peptides [#]	Conductance <t> [µs]	Flux V_{TM} [mV]	γ_{Na} [pS]	γ_{K} [pS]	γ_{Cl} [pS]	ANTS/DPX [(#/s)x10^6]	Water [#/s]
4a	4	4	400	0	0	0	-	3.0 x 10^7
5a	5	4	400	0	0	0	-	3.4 x 10^7
6a	6	4	400	0.1	0.4	1.0	-	1.1 x 10^9
7a	7	4	400	0.8	3.0	25.4	-	5.2 x 10^9
8a	8	4	100	0.9	1.7	21.6	-	1.4 x 10^{10}
8a	8	4	200	1.4	4.2	44.3	-	1.5 x 10^{10}
8a	8	4	400	15.2	23.6	293	-	1.7 x 10^{10}
8a†	8	4	0	-	-	-	0.0 / 1.0	0.9 x 10^{10}
8a†	8	4	100	0.7	1.2	16.7	0.5 / 0.8	1.2 x 10^{10}
8a†	8	4	200	1.7	3.1	34.7	1.0 / 1.0	1.4 x 10^{10}
8a†	8	4	400	16.4	19.7	223	63.0 / 12.0	1.6 x 10^{10}

Supplementary Table 2 | Conduction properties of spontaneously formed maculatin pores. Water flux and ionic conductance were determined at T = 37 °C by unbiased equilibrium simulations of single unrestrained pores in POPC bilayers in the presence of 500 mM NaCl and KCl. Each channel is only marginally stable and has its own characteristic architecture, oligomeric state, ion conductance and water and dye (ANTS/DPX) transport properties. Pores are shown in Figs. 3 and 4.

Supplementary Table 3 – Lifetimes of spontaneously formed maculatin pores

**Pore***	**Peptides** [#]	**Simulations** [#]	**<τ>** [ns]	**T** [°C]
6a	6	8	348±208	440
6a	6	8	209±103	450
6a	6	8	35±23	460
7a	7	8	165±146	440
7a	7	8	80±72	450
7a	7	8	39±31	460
7a	7	8	22±14	470
8a	8	8	1,292±526	410
8a	8	8	260±217	430
8a	8	8	79±54	450
8a	8	8	25±18	470

Supplementary Table 3 | Lifetimes of spontaneously formed maculatin pores. Functional pore lifetimes of spontaneously formed pores of high conductance were determined by 0.1-2.0 µs simulations of single unrestrained pores in POPC bilayers in the presence of 500 mM NaCl and KCl over a range of highly elevated temperatures. The unfolding kinetics were found to exhibit perfect Arrhenius behaviour, allowing estimation of pore lifetimes at lower temperatures (see Fig. 8).
